# Concentration-dependent activity of antibiotics in natural environments

**DOI:** 10.3389/fmicb.2013.00020

**Published:** 2013-02-13

**Authors:** Steve P. Bernier, Michael G. Surette

**Affiliations:** ^1^Farncombe Family Digestive Health Research Institute, Department of Medicine, Faculty of Health Sciences, McMaster UniversityHamilton, ON, Canada; ^2^Department of Biochemistry and Biomedical Sciences, Faculty of Health Sciences, McMaster UniversityHamilton, ON, Canada

**Keywords:** antibiotic, resistance, tolerance, interaction, signal, stress, cue, community

## Abstract

Bacterial responses to antibiotics are concentration-dependent. At high concentrations, antibiotics exhibit antimicrobial activities on susceptible cells, while subinhibitory concentrations induce diverse biological responses in bacteria. At non-lethal concentrations, bacteria may sense antibiotics as extracellular chemicals to trigger different cellular responses, which may include an altered antibiotic resistance/tolerance profile. In natural settings, microbes are typically in polymicrobial communities and antibiotic-mediated interactions between species may play a significant role in bacterial community structure and function. However, these aspects have not yet fully been explored at the community level. Here we discuss the different types of interactions mediated by antibiotics and non-antibiotic metabolites as a function of their concentrations and speculate on how these may amplify the overall antibiotic resistance/tolerance and the spread of antibiotic resistance determinants in a context of polymicrobial community.

## INTRODUCTION

Antibiotics are bioactive small molecules naturally produced by secondary metabolism of microorganisms such as bacteria and fungi (Davies, 2006; Aminov, 2010; Davies and Davies, 2010). Their discovery as antimicrobial drugs has revolutionized the management and treatment of infectious diseases. Many easily treated infectious diseases today had high mortality rates in the pre-antibiotic era. However, due to the increasing prevalence of resistance, the *in vivo* efficacy of antibiotics is reduced or abolished, and the spread of antibiotic-resistant microorganisms is now threatening the treatment of otherwise manageable infections.

Antibiotic resistance in microbes is widespread and it has been demonstrated that soil bacteria are rich in resistance determinants to both natural and synthetic antibiotics commonly used in clinics (D’Costa et al., 2006). The collective genes that contribute to antibiotic resistance are referred to as the antibiotic resistome (D’Costa et al., 2007; Wright, 2007). Interestingly, antibiotic resistance genes extracted from the soil resistome were shown to be identical or highly similar to those found in clinically relevant drug-resistant human pathogens (Forsberg et al., 2012) demonstrating that lateral gene transfer likely plays a role in the rise of multidrug-resistant pathogens. In addition to the environment, the human microbiome is also a niche rich in antibiotic resistance determinants where exchange of resistance genes can lead to the generation of drug-resistant bacteria with potential pathogenic traits (Sommer et al., 2009, 2010; Sommer and Dantas, 2011). Altogether, these recent studies showed that the spread of antibiotic resistance from non-pathogenic environmental bacterial is an ongoing threat to the clinical use of antibiotics, even for new synthetic compounds, and the emergence of new drug-resistant pathogens is a constant threat.

Despite a better understanding of the different mechanisms leading to resistance, exposure to antibiotics is still considered the major driver in the selection for antibiotic-resistant bacteria (Levy, 2001; Marshall and Levy, 2011; Andersson and Hughes, 2012) and the selection occurs over a large spectrum of concentrations (Andersson and Hughes, 2012). Lethal concentrations of antibiotics rarely occur outside of therapeutic applications, but bacteria constantly face subinhibitory antibiotics in the environment and the host (e.g., human and other animals) following therapies. In fact, the release of antibiotics in the environment from medical or non-medical (e.g., agricultural) use artificially creates concentration gradients that are rarely encountered by environmental bacteria located in areas that are normally free of human-derived antibiotic activities (Aminov, 2009; Martinez, 2009a). The rapid appearance of drug-resistant bacteria upon antibiotic exposure implies that resistance and resistance mechanisms have co-evolved with antibiotic-derived products. The latter point raises the question as to whether antibiotic resistance was already a bacterial trait before the modern use of antibiotics. To address this question, elegant metagenomic and functional studies showed the existence of resistance determinants in pristine areas of the world (D’Costa et al., 2011; Bhullar et al., 2012) demonstrating that antibiotic resistance predates the clinical use of antibiotics. Therefore, antibiotic resistance is a common bacterial feature. The medical and non-medical use of antibiotics may accelerate the spread of resistance through positive selection in both the environment and the host.

The focus on the medical use of antibiotics has limited fundamental research regarding the other potential activities of these compounds in their natural settings, including the environment (e.g., soil) and hosts such as humans, animals, and plants. In complex communities containing antibiotic-producing microorganisms, bacteria are naturally exposed to lethal and non-lethal antibiotics making them trained at responding to these compounds. Non-lethal levels of antibiotics can alter the expression of genes involved in a variety of bacterial functions like metabolism, regulation, virulence, DNA repair, and stress response (Goh et al., 2002; Tsui et al., 2004; Davies et al., 2006; Yim et al., 2006, 2007, 2011; Blazquez et al., 2012). Subinhibitory antibiotics can also modify cellular behaviors in bacteria with the formation of biofilms (Hoffman et al., 2005; Frank et al., 2007; Haddadin et al., 2010; Mirani and Jamil, 2011; Subrt et al., 2011; Kaplan et al., 2012) and persister cells (Dorr et al., 2010). Altogether, these observations strongly suggested that antibiotics induce responses other than those associated to their antimicrobial activities and it is now accepted that they might be used as “signaling” molecules with regulatory functions (Yim et al., 2007; Aminov, 2009; Allen et al., 2010).

Antibiotics are, like other bioactive small molecules, low molecular weight metabolites produced by secondary metabolism of microorganisms, i.e., are not considered essential for growth and viability. Microorganisms such as bacteria and fungi produce a wealth of small molecules that has been called the parvome (Davies, 2009; Davies and Ryan, 2012). Secondary metabolites are responsible for most of the interactions taking place in natural microbial communities (Lyon and Muir, 2003; Keller and Surette, 2006; Duan et al., 2009; Rath and Dorrestein, 2012) and as extracellular metabolites antibiotics have the potential to exhibit similar functions. Bacteria in natural environments are mostly part of complex polymicrobial communities in which all members share nutrient resources as well as chemicals including primary metabolic end products and secondary metabolites, which have the potential to induce antibiotic resistance/tolerance mechanisms. Natural communities also include host-associated communities such as the human microbiome. The role(s) of antibiotics in mediating non-lethal interactions between bacterial cells, may in fact, play a much bigger role than previously anticipated in the global antibiotic resistance threat that we are currently facing. Naturally occurring antibiotics, and other related secondary metabolites in bacterial communities, may dictate the spread of antibiotic resistance in a concentration-dependent manner. Therefore improving our current knowledge of antibiotic-mediated interactions in bacteria may facilitate the development of new therapeutic strategies for the treatment of drug-resistant pathogens. Here we review the current knowledge of antibiotic responsive activities as a function of their concentrations and speculate on how these antibiotic-mediated interactions may overall influence antibiotic resistance/tolerance and community composition in heterogeneous polymicrobial communities.

## CHEMICAL INTERACTIONS

The explosion of research in cell–cell interactions mediated by bioactive small molecules in microbiology has led to the general assumption that most interspecies cell–cell interactions could be labeled as “communication” or “signaling.” However, it is important to note that the demonstration of a biological response upon exposure to a chemical does not necessarily imply communication. The improper use of terms like signal, signaling, or communication in microbiology has created confusion since most interspecies metabolite-mediated interactions labeled as “signaling/communication” are often in conflict with evolutionary theories. A detailed analysis of the appropriate terminology is beyond the scope of this review; therefore we refer the reader to recent reviews that have thoroughly discussed the topic (Keller and Surette, 2006; Diggle et al., 2007; Stacy et al., 2012). A chemical mediating intra- or interspecies interactions can be defined as a signal, cue, or coercion (chemical manipulation). For a chemical interaction to occur, emitting bacteria must first produce a molecule that can be perceived by other individuals, and second, the receiver must alter its behavior in response to the signal.

To determine whether an interaction is mediated by a signal, a cue, or coercion the overall benefit of the reaction is used as primary criteria. As shown in **Table 1**, a signal is defined when both partners take advantage of the interaction (bidirectional), while cues or coercions have unidirectional benefits for receivers or emitters, respectively. In true signaling interactions, the production and detection of the signal have co-evolved specifically for that purpose and from an evolutionary perspective these events will only be maintained when both partners benefit from the information conveyed by the signal for which they evolved (Maynard Smith and Harper, 2003; Keller and Surette, 2006). On the other hand, a cue provides information to a receiver for which a response is triggered (Keller and Surette, 2006; Diggle et al., 2007; Stacy et al., 2012). Although not mediated by single molecules, environmental conditions can also be considered as cues by bacteria and they include pH, osmolarity, temperature, oxidative stress/oxygen, and nutrient limitation. The main distinction with a signal is that the biological response did not evolve for that purpose, which benefits only the receiver (Keller and Surette, 2006; Diggle et al., 2007; Stacy et al., 2012). Conversely, a coercion scenario is a strategy used by the emitter, via the release of a molecule, to chemically manipulate the receiver for its own benefit (Keller and Surette, 2006; Diggle et al., 2007; Stacy et al., 2012).

**Table 1:** Simplified description of chemical-mediated interactions^1,2^.

	Benefits the emitter	Benefits the receiver
Signal	++	+
Cue	-	+
Coercion	+	-

1 The overall benefit is used as the main determinant for the classification of the different types of bacteria–bacteria interactions and are either beneficial (+) or costly (-).

2 Adapted from Diggle et al. (2007) and Stacy et al. (2012).

**FIGURE 1:**
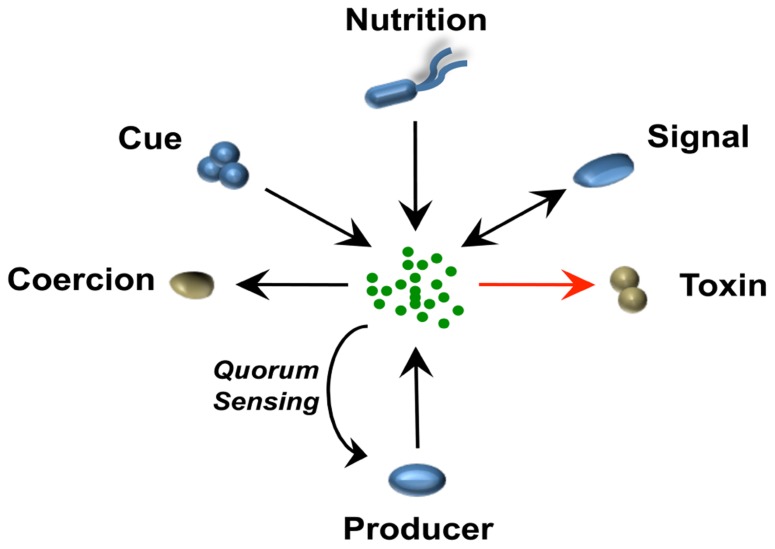
**A single chemical mediates different bacteria–bacteria interactions in a context of a polymicrobial community**. The schematic represents how a single chemical released by one bacterial species can be perceived differently in a multispecies community. Microorganisms that benefit positively (blue) or negatively (brown) from the different interaction are represented. Arrows indicate direction of evolved pathway, e.g., an organism evolves to “sense” a cue or in some case can use an antibiotic as a nutritional source (Dantas et al., 2008). In the case of signaling and interspecies signaling, pathways from both producer and receiver cells have co-evolved to the benefit of both microorganisms. In the case of coercion or toxic interactions such as those exhibited by antibiotics, only the producer benefits from the interaction. When the producer itself detects the signal, this would be a case of quorum sensing or cell–cell signaling. In the case of antibiotics, the producer usually co-expresses resistance pathways and these can be co-expressed without a sensing circuit but often, such as with many lantibiotics, they are detected by the cell and respond in a classical quorum sensing feedback loop.

**Figure 1** illustrates the different ways by which a single chemical can be perceived by bacterial cells of different species within a polymicrobial community. Although interactions mediated by signals, cues, or coercions can all occur in these communities, the majority of these dynamic interactions fall in the category of cue, because they do not require stability over time, for which only receiver cells evolve. The modulation of *Pseudomonas aeruginosa* virulence factors by autoinducer-2 (AI-2) from the oropharyngeal microbiota (Duan et al., 2003) and alteration of the antibiotic tolerance profile by volatile ammonia (Bernier et al., 2011) are examples of interspecies interactions mediated by cues.

Interestingly, intraspecies diversity may in some cases challenge the concept of true communication by the rise of cheaters through genetic mutations. The inability of cheaters to either produce and/or perceive a signal may abolish the bidirectional cooperative interaction. In a situation where the response toward a signal leads to the production of a protease allowing the degradation of a particular substrate for nutritional purposes, cheaters impaired in either the production or the reception of a signal will differentially impact the cooperative interaction. In fact, cheaters get a direct competitive advantage by avoiding the metabolic cost of producing a signal or responding to it and their selfish behavior allows them to benefit without being cooperative. However, some systems may have co-regulated pathways that help to control cheaters (Dandekar et al., 2012).

## BIOACTIVITY AND ANTIBIOTIC RESISTANCE ARE DRIVEN BY ANTIBIOTIC CONCENTRATIONS

Antibiotics are generally known for their antimicrobial properties by which they either kill (bactericidal) or inhibit bacterial growth (bacteriostatic). Their concentrations are highly variable in natural communities and bacteria have evolved mechanisms to respond accordingly. Although their antimicrobial properties have been demonstrated in both *in vitro* and *in vivo *settings, the biological roles of antibiotics and antibiotic resistance in natural environments are still undefined.

**FIGURE 2:**
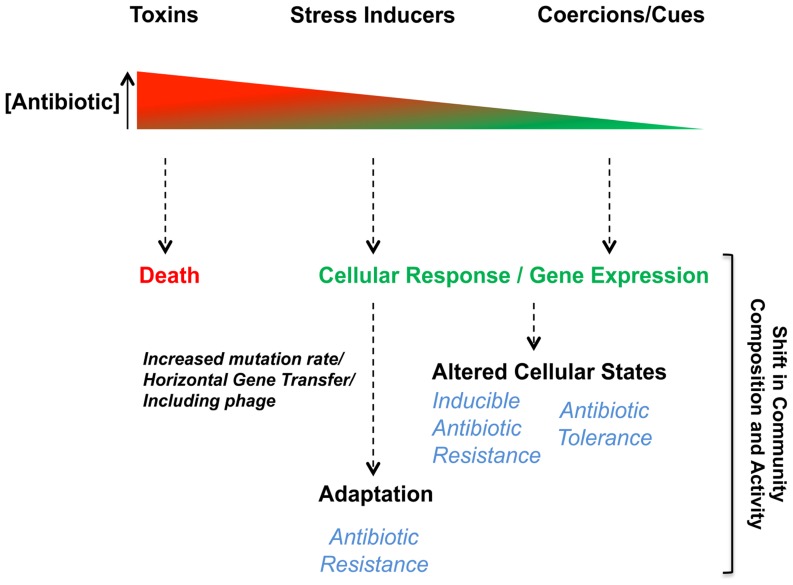
**Biological responses toward antibiotics are concentration-dependent**. Bacterial interactions mediated by antibiotics induce biological responses in receiver bacteria in a dose-dependent manner. The antimicrobial behavior (toxin) of antibiotics occurs when their concentrations is high leading to bacterial death or growth arrest in susceptible receiver cells. At lower concentrations, subinhibitory, antibiotics can act as stress inducers, coercions or be sensed as cues. Biological responses induced in receiver bacteria when antibiotics are at subinhibitory concentrations can affect various cellular responses or alter gene expression leading to different adaptive responses impacting antibiotic resistance/tolerance.

To first differentiate the biological responses induced by antibiotics, here we represent their bioactivities from a receiver bacterium’s perspective on a large concentration spectrum (**Figure 2**). From high to low concentrations, antibiotics act as either toxins, stress inducers, or as cues/coercions, respectively. Among interactions mediated by subinhibitory antibiotics, receiver bacteria can interestingly induce mechanisms leading to antibiotic resistance or tolerance (**Figure 2**). How antibiotics become stress inducers or cues/coercions will further be discussed in the following sections, but before we will briefly describe the toxin-like behavior of antibiotics and the impact of antibiotic resistance on the biological response exhibited by bacteria upon antibiotic exposure.

At concentrations superior to or near the minimal inhibitory concentration (MIC), antibiotics behave like toxins on susceptible bacterial cells. However, although the understanding of resistance mechanisms is well-characterized, molecular mechanism(s) induced by lethal concentrations of antibiotics is an area of research where our fundamental understanding is still very limited (Kohanski et al., 2010). Recent studies suggested that antibiotic-induced cell death was associated with increased production of radical oxygen species (ROS) such as hydroxyl radicals, superoxide, and hydrogen peroxide for bactericidal antibiotics belonging to the families of quinolones, β-lactams, and aminoglycosides (Dwyer et al., 2007, 2009; Kohanski et al., 2007, 2008, 2010). Although antibiotic ROS-mediated killing is highly possible in aerobic conditions, the proposed model is however, oxygen-dependent (Hassett and Imlay, 2007) and other mechanisms must operate in oxygen-poor environments such as those found in biofilm populations (Stewart and Franklin, 2008).

**FIGURE 3:**
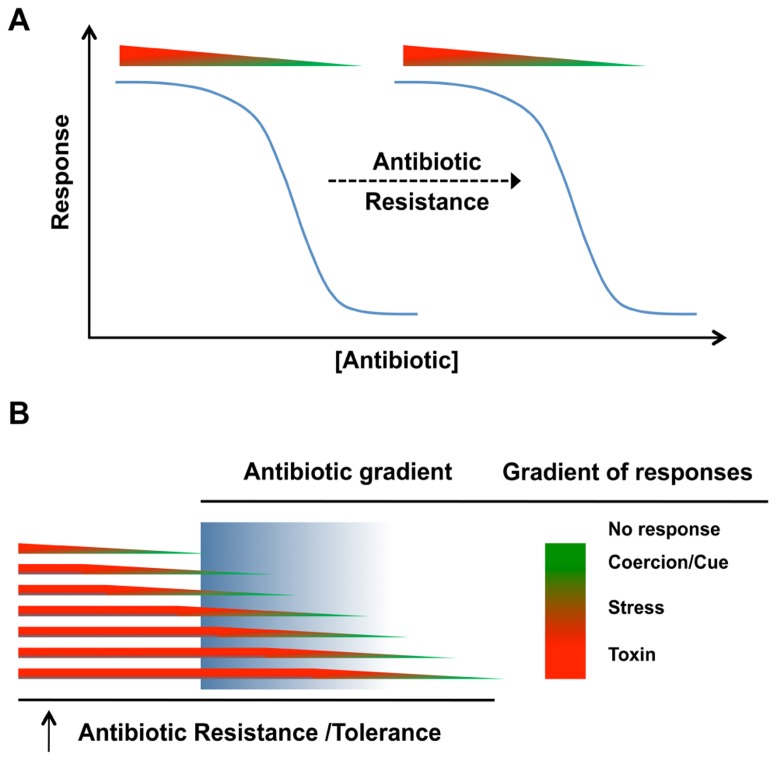
**Biological role(s) of antibiotic resistance in natural communities**. **(A) **The response of bacterial cells to antibiotic whether it is bactericidal/bacteriostatic or subinhibitory, the biological response (as a cue or coercion) will be shifted by antibiotic resistance. **(B)** In natural environments, differences in physiological states can impact the expression of antibiotic resistance or tolerance mechanisms. Antibiotic concentrations in these environments may also be distributed as gradients, meaning that the population will not necessarily respond uniformly.

Antibiotic resistance mechanisms and the antimicrobial nature of antibiotics are often associated as a cause and effect phenomenon. The presence of resistance genes in bacteria with antibiotic biosynthesis genetic loci (Benveniste and Davies, 1973; Walker and Skorvaga, 1973; Davies and Benveniste, 1974) is an obvious self-protective strategy (Martinez, 2009a,b; Davies and Ryan, 2012; Wright and Poinar, 2012). It has therefore been widely accepted that antibiotic resistance determinants have specifically evolved to tolerate the lethal activity of antibiotics (Martinez, 2009a,b), but experimental data to fully support this thesis are still lacking (Davies and Ryan, 2012). Antibiotics are mainly present at non-lethal concentrations in the environment (Martinez, 2009a,b), therefore antibiotic resistance determinants are likely involved in response mechanisms other than those required when receiver bacteria are exposed to lethal concentrations. Similar to the antibiotic concentration-dependent response (**Figure 2**), antibiotic resistance would also impact receiver bacteria in an antibiotic dose-dependent manner. The presence of an antibiotic resistance mechanism would shift the spectrum of responses to an antibiotic to higher concentrations. The resistance would lower the effective concentration of antibiotics at the target site (**Figure 3A**). Exceptions to this would occur if there were secondary target sites for the antibiotics that mediate other responses. At toxic concentrations, resistance would function in the conventional protective role and allow receiver bacteria to avoid the antibiotic toxicity by blocking death or growth arrest. At subinhibitory concentrations, antibiotic resistance genes would shift the effective antibiotic concentration required for inducing the biological responses (stress inducers, coercion, and cues) of receiver bacteria (Goh et al., 2002; Yim et al., 2007; Mesak et al., 2008; Mesak and Davies, 2009). The displacement of the response curve to subinhibitory concentrations of antibiotics due to the presence of resistance determinants (**Figure 3A**) could therefore establish a chemical “*arms race*” between producer and receiver bacteria independent of lethal effect. In the context of bacterial communities, the displacement of the response curve for a single strain will modify its own behavior, which may in return shift the community composition or activity. Stress induction, coercion, and detection of cues are selectable phenotypes that can be tuned to meet the conditions of natural environments. Selections that reduce stress induction and coercion could be contributing factors in the evolution of the “cryptic resistome” (Wright, 2007); genes that are normally expressed at low levels or have low specific activity that do not confer resistance to the toxic effects of antibiotics at higher concentrations. These may be resistance genes tuned to environments where there are lower concentrations of these bioactive molecules.

In natural environments, antibiotics are likely present in a gradient of concentrations while receiver organisms may also be heterogeneous. Even in a homogeneous clonal population, cells may be present in different physiological states and the presence of antibiotic tolerance and resistance mechanisms will shift the response curves for individual cells within the population (**Figure 3B**). In the context of chemical interactions within natural communities, antibiotics released by emitter bacteria at lethal concentrations for the receiver cells would not be considered as a signaling event, but rather coercion at the extreme. Coercion can occur at subinhibitory concentrations and one bacterial cell may use chemicals to manipulate another. If the receiver cell possesses the corresponding antibiotic resistance determinant, receiver bacteria will be less susceptible to chemical manipulation and the overall benefit or harm of the interaction will therefore be reduced.

Outside of the well-mixed homogeneous environments of laboratory cultures, antibiotic effects on bacterial populations will be heterogeneous. For any bacteria, the response to a specific antibiotic will be concentration-dependent (**Figure 2**) and the active concentration for a particular response will be shifted higher by resistance mechanisms (**Figure 3A**). In natural environments, a population will be expected to exhibit a heterogeneous response because of gradients of antibiotic concentration and heterogeneity in the responsiveness of different cells in the population (**Figure 3B**). These differences in cellular responsiveness may be due to genetic heterogeneity and/or through differences in physiological states of different cells in the population.

## ANTIBIOTICS AS STRESS INDUCERS

Antibiotics at subinhibitory concentrations can act as stress inducers or cues/coercion on receiver bacteria (**Figure 2**). When behaving as stress inducers, antibiotics often induce the SOS stress response, which is also associated with various antibiotic resistance mechanisms. The following section will mainly highlight some of the main resistance mechanisms impacted by the induction of the SOS response in bacteria upon antibiotic exposure.

Bacteria possess multiple survival mechanisms to cope with exogenous stresses and the SOS response is the main general stress response induced by bacteria in these situations (Galhardo et al., 2007; Blazquez et al., 2012; Poole, 2012b). The SOS stress response is typically induced upon DNA damage caused by extracellular stresses such as bacterial cell exposure to UV light or antibiotics (Erill et al., 2007; Janion, 2008; Butala et al., 2009). It is characterized by a well-coordinated global response initiating inhibition of cell division and induction of DNA repair, recombination, and mutation (Erill et al., 2007; Janion, 2008; Butala et al., 2009). In most bacterial species, the RecA and LexA proteins govern the response, which is conserved across bacterial phyla with a few exceptions where the LexA repressor protein homolog is absent, such as in *Streptococcus* species (Erill et al., 2007). Upon DNA damage, RecA stimulates cleavage of the LexA repressor leading to the global response involving more than 40 SOS-regulated genes (Courcelle et al., 2001; Erill et al., 2007; Janion, 2008; Butala et al., 2009). For a more comprehensive description of the SOS stress response, we refer the reader to consult some of these reviews that have thoroughly discussed the topic (Erill et al., 2007; Janion, 2008; Butala et al., 2009).

Fluoroquinolones and quinolones are broad spectrum antibiotics, for which resistant mechanisms have quickly emerged (Ruiz, 2003). They inhibit DNA gyrase leading to double-stranded DNA breaks and consequently induction of the SOS stress response (Urios et al., 1991; Dwyer et al., 2007; Yim et al., 2011). This induction usually occurs within a particular window of antibiotic concentrations (Piddock and Wise, 1987). In addition to fluoroquinolones or quinolones, bactericidal β-lactam and aminoglycoside antibiotics mediate bacterial killing by stimulating the production of ROS (Dwyer et al., 2007, 2009; Kohanski et al., 2007, 2008, 2010), which are themselves potent DNA damaging molecules (Farr and Kogoma, 1991). Consequently, all antibiotics mediating the production of ROS would therefore have the potential to induce the SOS stress response. Interestingly, fluoroquinolone and β-lactam antibiotics were shown in multiple studies to induce the SOS stress response in *Escherichia coli *while aminoglycosides failed (Ysern et al., 1990; Miller et al., 2004; Baharoglu and Mazel, 2011; Poole, 2012b). The intimate relationship between ROS-mediated killing and SOS-induction by bactericidal antibiotics requires more investigations to explain these discrepancies, but it does suggest that the stimulation of ROS production upon antibiotic exposure may be an indirect effect. Parallel mechanisms to the SOS response may exist for aminoglycosides to kill bacteria in a ROS-dependent manner or concentrations must be lethal to induce the SOS response and not subinhibitory. Other antibiotic classes represented by trimethoprim, ceftazidime, and sulfamethoxazole are also strong inducers of the SOS stress response in *E. coli* (Blazquez et al., 2012).

The SOS stress response is widespread among bacteria (Erill et al., 2007), but differences in the antibiotic SOS-induction profiles have been observed between species. For example, subinhibitory concentrations of tetracycline, chloramphenicol, and aminoglycosides induce the SOS response of *Vibrio cholerae*, while these antibiotics have no impact in *E. coli* (Baharoglu and Mazel, 2011). Despite the strong similarities between SOS systems across Gram-negative bacteria and the genetic relatedness of *E. coli* and *V. cholerae*, these disparities suggest that antibiotics may not necessarily induce the SOS response directly from DNA damage, but rather via upstream pathways or targets of the SOS response (Aertsen and Michiels, 2006) that could potentially differ between bacterial species. These differences between bacterial species may reflect the evolutionary selective pressure on the different bacteria with specific features reflecting conditions of their natural environments.

The induction of the SOS response is often essential for bacterial survival in stressful environments and associated with genetic responses that indirectly alter antibiotic resistance by increasing mutation rate, horizontal gene transfer, and prophage induction (Erill et al., 2007; Janion, 2008; Butala et al., 2009; Dwyer et al., 2009). Several studies have shown that induction of the SOS response by various antibiotics like fluoroquinolones, β-lactams, trimethoprim, tetracycline, chloramphenicol, rifampicin, and aminoglycosides increased the mutation frequency in different bacterial species (Ysern et al., 1990; Gillespie et al., 2005; Henderson-Begg et al., 2006; Cortes et al., 2008; Mesak and Davies, 2009; Baharoglu and Mazel, 2011; Yim et al., 2011). Induction of competency by fluoroquinolones leading to horizontal gene transfer with the potential to acquire new antibiotic resistance determinants has recently been reviewed (Charpentier et al., 2012). Furthermore, antibiotics inducing the SOS response can promote bacterial genetic diversity via homologous recombination, phage release, and transfer of integrons or conjugative elements (Matic et al., 1995; Beaber et al., 2004; Cirz et al., 2007; Hocquet et al., 2012) all involved in the movement of mobile DNA like antibiotic resistance genes.

The induction of the SOS response is critical and relevant to our understanding of antibiotic-mediated interactions on the overall impact of antibiotic resistance in bacterial populations. Bacteria in most environments including those affected during therapeutic treatments can potentially encounter subinhibitory concentrations of antibiotics inducing the SOS stress response. Therefore, a better understanding of SOS-associated behaviors linked to antibiotic resistance traits may result in alternative approaches to control their spread.

## ANTIBIOTICS AS CUES

Various antibiotics at subinhibitory concentrations induce biological responses on receiver bacteria that are non-stress-related and frequently affect pathways of primary metabolism (Goh et al., 2002; Tsui et al., 2004; Davies et al., 2006; Yim et al., 2006, 2007, 2011; Blazquez et al., 2012). Here we highlight examples demonstrating bacterial interactions mediated by antibiotics sensed as cues, which can subsequently impact the antibiotic resistance profile of receiver bacteria. These inducible responses can directly target mechanisms leading to specific antibiotic resistance or indirectly impact tolerance toward various antibiotics.

Many bacteria can sense specific antibiotics in their environment and subsequently induce the corresponding resistance mechanisms. Tetracycline and vancomycin are the two best-studied examples. Tetracycline resistance has been attributed to classical resistance mechanisms including efflux strategies, target site access (TetM and TetO), and chemical inactivation (TetX; Nelson and Levy, 2011). The regulation of some tetracycline resistance determinants is under the control of tetracycline repressor protein (TetR), which has been thoroughly reviewed elsewhere (Hillen and Berens, 1994; Berens and Hillen, 2003; Ramos et al., 2005; Nelson and Levy, 2011). Briefly, TetR has DNA binding domains targeting operators of tetracycline resistance genes. Once bound to the operator region of a target gene, TetR acts as a transcriptional repressor for which the repression can be relieved by the interaction of tetracycline with TetR (Hillen and Berens, 1994; Berens and Hillen, 2003; Ramos et al., 2005). Therefore, when tetracycline is present, the repressive function of TetR is abolished and transcription of the tetracycline resistance gene can occur normally.

In the case of vancomycin, modification of the target (peptidoglycan – D-Ala-D-Ala C-terminus) is the primary mechanism of resistance (Courvalin, 2006). Six types of resistance to vancomycin (VanA, B, C, D, E, and G) were reported in *Enterococcus* species (Courvalin, 2006). Interestingly, four of these operons involved in vancomycin resistance (VanA, B, E, and G) are directly inducible by vancomycin, while VanG and C types are constitutive (Courvalin, 2006). Vancomycin is sensed by a two-component regulatory system that positively activates expression of resistance genes in response to vancomycin. Although the basic regulation differs between tetracycline and vancomycin resistance genes, the inducible nature of their specific resistance pathways demonstrate that antibiotics have the ability to directly induce targeted and specific resistance mechanisms.

**FIGURE 4:**
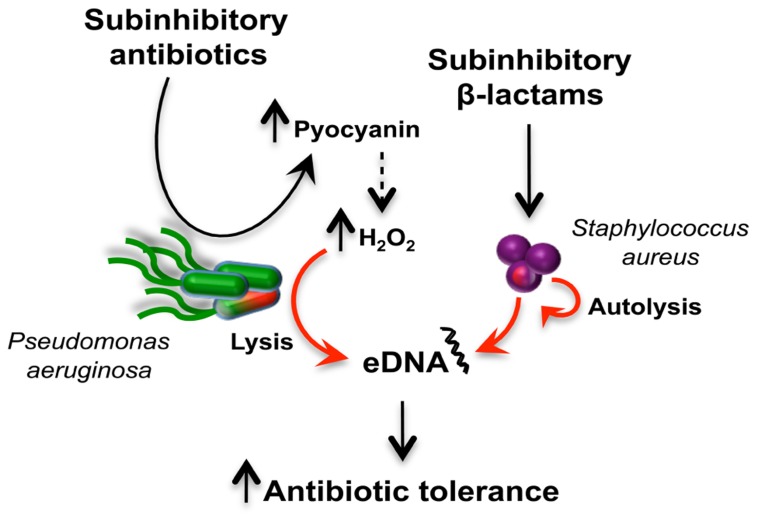
**Antibiotic induction of extracellular DNA (eDNA) release in a multispecies community and antibiotic tolerance**. The schematic represents how two bacterial species, *P. aeruginosa* (green) and *S. aureus* (purple), respond to subinhibitory antibiotics to release eDNA. Subinhibitory antibiotics induced the production of pyocyanin (Shen et al., 2008), which is associated with increased H_2_O_2_ levels responsible for cell lysis and DNA release (Das and Manefield, 2012). In *S. arureus*, exposure to subinhibitory β-lactam antibiotics induces release of eDNA via an autolysis-dependent mechanism (Kaplan et al., 2012). The release of eDNA by both bacteria can induce antibiotic tolerance in communities (Mulcahy et al., 2008).

Although subinhibitory concentrations of antibiotics like tetracycline and vancomycin can induce their own resistance mechanisms, other biological responses have the potential to indirectly impact antibiotic tolerance as well. It was recently demonstrated that subinhibitory concentrations of β-lactam antibiotics induce the autolysin-dependent release of extracellular DNA (eDNA) by *Staphylococcus aureus*. This affects biofilm formation and autoaggregation (Kaplan et al., 2012), two growth protective mechanisms that will be discussed in the following section. Interestingly, eDNA that is part of the extracellular matrix of *P. aeruginosa* biofilms induces tolerance against aminoglycosides by chelating cations (Mulcahy et al., 2008). Additionally, subinhibitory concentrations of antibiotics like vancomycin, tetracycline, azithromycin, and ampicillin induce the expression of *P. aeruginosa* virulence-associated genes leading to increased secretion of phenazines and rhamnolipids (Shen et al., 2008). Pyocyanin, one of the four *P. aeruginosa* phenazines (Price-Whelan et al., 2006) was recently showed to induce eDNA release in *P. aeruginosa* biofilms through hydrogen peroxide (H_2_O_2_) mediating cell lysis (Das and Manefield, 2012). These examples provide evidence that in multispecies communities, subinhibitory antibiotics can lead to a series of sequential responses leading to antibiotic tolerance (**Figure 4**).

These are a few examples demonstrating that sensing of subinhibitory concentrations of antibiotics as cues can trigger direct or indirect mechanisms, for which receiver bacteria will subsequently have the ability to resist or tolerate lethal concentrations of antibiotics. In microbial communities, the response of one organism may lead to induction of antibiotic resistance/tolerance in other bacteria.

## ANTIBIOTICS AS INDUCERS OF GROWTH PROTECTIVE MECHANISMS

A significant portion of the antibiotic resistome is made of mobile resistance genes that are horizontally transferable between bacterial cells (D’Costa et al., 2007; Wright, 2007; Allen et al., 2010). Mobile resistance determinants as well as efflux pumps account for the majority of antibiotic resistance mechanisms, however, bacteria have evolved non-inherited and transient mechanisms to resist otherwise lethal antibiotic concentrations (Levin and Rozen, 2006). Environmental conditions can trigger various stress responses making bacterial cells transiently refractory to antibiotics. Bacterial stress responses as determinants of antibiotic resistance have become an emerging area of research. For a more comprehensive description of these we refer the reader to recent reviews that have thoroughly addressed the topic (Poole, 2012a,b). Briefly, bacterial exposures to environmental-related stresses like nutrient starvation/limitation (nutrient stress), ROS and reactive nitrogen species (oxidative/nitrosative stress), membrane damage (envelope stress), temperature (heat/cold stress), and ribosome disruption (ribosomal stress) have the potential to initiate bacterial responses leading to modified or enhanced tolerance toward the lethal action of antibiotics (Poole, 2012a,b).

Non-inherited and transient mechanisms are mainly attributed to two distinct processes: persistence and drug indifference (Levin and Rozen, 2006). While persistence occurs in subpopulations of slow or non-growing bacteria, drug indifference can be exhibited by the entire population (Levin and Rozen, 2006). Persister cells are generally considered to be responsible for bacterial survival following antibiotic treatments although heterogeneity within bacterial populations and reduced accessibility of the drug to some target cells also contribute. A comprehensive description of persister cells is beyond the scope of this review; therefore we refer the reader to recent reviews that have thoroughly discussed the topic (Lewis, 2007, 2010, 2012; Gerdes and Maisonneuve, 2012; Kint et al., 2012). Persisters are phenotypic variants within an isogenic bacterial population that can tolerate high concentrations of antibiotics. In contrast to drug-resistant cells, persisters can switch back to the wild-type antibiotic sensitive phenotype when reactivated (Lewis, 2007, 2010, 2012; Gerdes and Maisonneuve, 2012; Kint et al., 2012). This persister cell behavior has been directly observed (Balaban et al., 2004). Persisters are present both in planktonic bacteria and antibiotic-tolerant biofilms (Spoering and Lewis, 2001). Research emphasis in the field has largely focused on mechanisms involved in persister formation and mechanisms leading to their formation have been proposed such as stochastic processes (passive) to active inducible regulation (Kint et al., 2012). Stochastic switching has been proposed as an effective strategy for survival in unpredictable environments (Kussell and Leibler, 2005; Kussell et al., 2005). Dormancy is a passive mechanism involved in persister formation resulting from stochastic endogenous stress leading to growth arrest and the shutdown of bactericidal antibiotic targets making persisters multidrug-tolerant cells (Lewis, 2007, 2010, 2012). Experimental evidence to support the dormancy theory came from transcriptome analysis showing that genes involved in primary metabolism and energy production were down-regulated in persister cells (Keren et al., 2004; Shah et al., 2006). However, the dormancy model was challenge when persisters of *E. coli* were demonstrated to display some level of protein translation (Gefen et al., 2008).

Active mechanisms leading to the induction of persisters in bacterial populations differ in terms of target. After the identification of *hipA*, as the first persistence gene and coding for the toxin of the *hipAB* toxin–antitoxin module (TA; Moyed and Bertrand, 1983), many studies have shown that different TA modules were involved in bacterial persistence (Lewis, 2010, 2012; Gerdes and Maisonneuve, 2012; Kint et al., 2012). The induction of toxin genes in persister cells (Keren et al., 2004; Shah et al., 2006) or the overexpression of toxins leading to increased persistence (Keren et al., 2004; Shah et al., 2006; Harrison et al., 2009; Maisonneuve et al., 2011) supported the role of TA modules in bacterial persistence. Interestingly, the fluoroquinolone ciprofloxacin was shown to induce bacteria persistence via the TA module TisAB upon activation of the SOS response (Dorr et al., 2010). Consistently, lethal concentrations of ampicillin or ofloxacin induce the SOS stress response in persister cells (Kaldalu et al., 2004) while the SOS response confers persistence to fluoroquinolones (Dorr et al., 2009). Unrelated to TA modules, the extracellular chemical indole was recently shown as mechanism inducing persisters in *E. coli* populations (Vega et al., 2012). Interestingly, the latter represents a non-antibiotic mediated interaction leading to antibiotic tolerance; a topic that will be discussed in the following section.

Beside unicellular growth, bacteria can also adopt various types of multicellular growth that exhibit phenotypes different than their planktonic counterparts including antibiotic tolerance. Multicellular behaviors in bacteria include growth as biofilms, aggregates, and swarming. Biofilms are sessile bacterial cells encased within an extracellular matrix that are usually attached to a surface (Stewart and Franklin, 2008; Monds and O’Toole, 2009). Bacterial aggregates are described as unattached biofilm-like structures with the ability to move (Alhede et al., 2011; Haaber et al., 2012; Thornton et al., 2012) while swarming represents a type of motility exhibited by bacteria over semi-solid surfaces (Kearns, 2010). These multicellular behaviors were all associated with elevated tolerance to lethal concentrations of antibiotics when compared to their planktonic counterparts (Stewart and Costerton, 2001; Kim et al., 2003; Lewis, 2007; Overhage et al., 2008; Lai et al., 2009; Alhede et al., 2011; Haaber et al., 2012; Thornton et al., 2012). These studies demonstrate that multicellular assemblies in bacteria confer an advantage when facing antibiotics compared to planktonic cells. However, the growth phase of planktonic cells (i.e., logarithmic or stationary) can have a significant impact on their antibiotic tolerance profiles. For example, planktonic *P. aeruginosa* and *E. coli* cells were previously shown to exhibit greater levels of tolerance against bactericidal antibiotics (Evans et al., 1991; Spoering and Lewis, 2001; Bernier et al., 2013). In *Salmonella typhimurium* shifts in primary metabolic pathways have been associated with the induced antibiotic tolerance in swarm cells (Kim and Surette, 2003, 2004; Turnbull and Surette, 2008, 2010) and aggregates (White et al., 2010).

Antibiotic-mediated interactions impact multicellular behaviors and indirectly the antibiotic tolerance profile of these populations. Several studies have showed that subinhibitory antibiotics induce biofilm formation (Hoffman et al., 2005; Frank et al., 2007; Haddadin et al., 2010; Mirani and Jamil, 2011; Subrt et al., 2011; Kaplan et al., 2012) and autoaggregation (Kaplan et al., 2012). Bacterial response to extracellular stresses (Poole, 2012a,b) may also be an important trigger of multicellular behaviors in bacteria (Kaplan, 2011), which are better adapted as a group due to their physiology to tolerate lethal antibiotics (Stewart and Franklin, 2008). In support of this hypothesis, a recent study showed that non-starving planktonic cells were generally more tolerant to bactericidal antibiotics than biofilms, but when these same bacterial cells were starved, therefore stressed, biofilm bacteria were significantly more resilient to antibiotics than their planktonic counterparts (Bernier et al., 2013). As one of the first responses to stress, the SOS response is significantly more induced in biofilm cells compared to their planktonic counterparts (Beloin et al., 2004; Bernier et al., 2013). The higher intrinsic level of SOS in biofilms may explain their increased mutation frequency compared to planktonic cells (Conibear et al., 2009). Altogether, the increased SOS-dependent mutation rate observed in biofilms may well explain the high level of genetic variants arising in biofilm populations in a RecA-dependent manner (Boles et al., 2004; van der Veen and Abee, 2011) with the potential to impact antibiotic resistance (Boles and Singh, 2008).

The unique physiology of multicellular behaviors such as biofilms and swarming bacteria may render these cells better adapted to respond and tolerate extracellular stresses such as otherwise lethal antibiotic concentrations, these states may be induced directly by subinhibitory antibiotics.

## NON-ANTIBIOTIC SMALL MOLECULES AS MODULATORS OF ANTIBIOTIC TOLERANCE

We have highlighted previous studies demonstrating that bacteria can sense antibiotics as cues to mediate bacteria–bacteria interactions with the potential to induce resistance/tolerance when lethal concentrations are subsequently reached. These can induce antibiotic specific mechanisms (e.g., tetracycline, vancomycin) or more general mechanisms like biofilms induction. However, the induction of antibiotic resistance/tolerance via cues is not limited to antibiotics. Within natural communities, bacteria are continuously exposed to a variety of small molecules other than antibiotics. Among these bioactive metabolites, some have been shown to induce biological responses in bacteria leading to a change in the overall antibiotic tolerance profile.

Bacterial-derived extracellular metabolites such as indole, hydrogen sulfide (H_2_S), and volatile ammonia were recently shown to impact the antibiotic tolerance profile of receiver bacteria (Lee et al., 2010; Bernier et al., 2011; Shatalin et al., 2011; Vega et al., 2012). Interestingly, all of these bioactive molecules have the ability to be soluble or volatile, however, only ammonia was studied under its gaseous phase (Bernier et al., 2011).

Indole, a tryptophan-derived aromatic heterocyclic organic compound, was recently reported to induce antibiotic resistance in *E. coli* (Lee et al., 2010; Vega et al., 2012). A community-based antibiotic resistance mechanism was demonstrated to occur via the release of the metabolite in continuous cultures of *E. coli* exposed to increasing levels of the norfloxacin quinolone (Lee et al., 2010). Briefly, under antibiotic stress, a few drug-resistant mutants arise and then release the metabolite indole that is sensed by the entire population allowing other less resistant isolates to survive (Lee et al., 2010). In this particular case, the overall population MIC is totally biased by a few resistant clones since the majority of isolates are sensitive (Lee et al., 2010). The altruistic behavior of drug-resistant isolates comes with a fitness cost, associated with the production of indole, that benefits the entire population (Lee et al., 2010). Increased antibiotic tolerance mediated by indole is proposed to result from induction of efflux pumps and oxidative stress protective mechanisms (Lee et al., 2010). In a different study, indole is proposed to directly induce persistence through the generation of persister cells (Vega et al., 2012). The generation of persisters by indole exposure is dependent on the phage-shock (Psp) and OxyR pathways (Vega et al., 2012). In other studies, indole was shown to promote the establishment of *E. coli* in dual-species cultures with *P. aeruginosa* by inhibiting production of pyocyanin and other *P. aeruginosa* virulence factors regulated by quorum sensing (Chu et al., 2012). This example represents an example of coercion in which *E. coli*-derived indole manipulates *P. aeruginosa* as a strategy to colonize and share a polymicrobial community. Conversely, over production of indole through the induction of ROS production by subinhibitory concentrations of antibiotics was shown to impair *E. coli* biofilm formation (Kuczynska-Wisnik et al., 2010).

Until recently, the production of extracellular H_2_S by bacteria has mainly been considered as a toxic by-product of metabolism. However, bacterial-derived H_2_S is protective against the lethal action of antibiotics in a ROS-dependent manner (Shatalin et al., 2011). It was also demonstrated that endogenous nitric oxide (NO) of Gram-positive bacteria was protective against oxidative stress-mediated killing by macrophages (Shatalin et al., 2008) and antibiotics (Gusarov et al., 2009). Interestingly, NO and H_2_S act synergistically since the absence of one can be compensated for by the increased production of the other one upon antibiotic exposure (Shatalin et al., 2011). These studies demonstrate the biological relevance of bacterial gases in mediating bacterial interactions and their indirect impact on antibiotic resistance.

Ammonia, a general by-product of amino acid catabolism, was recently shown to modulate the antibiotic tolerance of neighboring bacterial cells (Bernier et al., 2011). Briefly, both Gram-negative and Gram-positive bacterial species were able to tolerate otherwise lethal concentrations of ampicillin and tetracycline upon exposure to biogenic volatile ammonia (Bernier et al., 2011). Conversely, sensitivity toward aminoglycoside antibiotics was increased in bacterial cells exposed to volatile ammonia (Bernier et al., 2011). Ammonia-mediated interactions between bacterial cells were shown to induce the intracellular levels of polyamines (Bernier et al., 2011). Consistently, addition of polyamines (spermidine and putrescine) could recapitulate the ammonia-mediated phenotype demonstrating that the modified antibiotic tolerance profile of receiver bacteria was fully dependent on the polyamine modulon upon ammonia exposure (Bernier et al., 2011). Interestingly, it was demonstrated in *Bacillus* spp. that biofilm formation could also be induced upon exposure to biogenic ammonia and polyamines were critical for normal biofilm development (Burrell et al., 2010; Nijland and Burgess, 2010).

The close relationship between ammonia sensing, polyamine induction, and biofilm formation demonstrate that bacterial interactions mediated by non-antibiotic molecules can modulate antibiotic tolerance not only locally, but also at a distance when ammonia is under its gaseous phase. Altogether, non-antibiotic-mediated interactions clearly demonstrate the complexity of dealing with antibiotic resistance/tolerance when bacteria are part of complex communities, making antibiotic treatments unpredictable.

## METABOLITE-MEDIATED INTERACTIONS IN BACTERIAL COMMUNITIES AS MECHANISMS OF ANTIBIOTIC RESISTANCE EVOLUTION

Many infections are polymicrobial and as presented above, antibiotics and non-antibiotic metabolites can mediate interactions between organisms that may impact their efficacy as antimicrobials. The following describes a hypothetical scenario demonstrating how metabolite-mediated interactions may have the potential to alter the antibiotic resistance profile of an entire population and possibly leading to the spread of antibiotic resistance.

**FIGURE 5:**
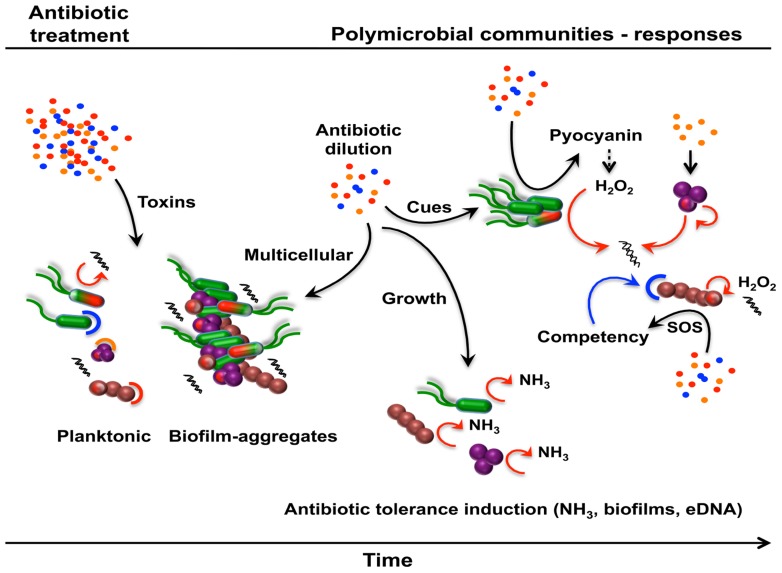
**Metabolite-mediated interactions modulating antibiotic resistance or tolerance in polymicrobial communities**. Following an antibiotic treatment, natural communities will respond, adapt, and evolve through the establishment of dynamic interactions mediated by antibiotics and non-antibiotic metabolites. A simple polymicrobial community comprising *P. aeruginosa* (green), *S. aureus* (purple), and *Streptococcu*s spp. (brown) is used to illustrate this hypothetical scenario. A proportion of the established community, made of multispecies biofilms and planktonic cells, will die and subsequently lyse upon antibiotic exposure, unless cells harbor the corresponding antibiotic resistance determinant (capped cells; red, blue, or orange corresponding to the respective antibiotic) or are part of multicellular structures (biofilms, aggregates). Subinhibitory antibiotics and non-antibiotic metabolites within the community can subsequently induce different antibiotic tolerance mechanisms leading to an altered community in terms of composition and antibiotic resistance/tolerance.

For this exercise, we chose a simple polymicrobial community including the pathogens *P. aeruginosa* and *S. aureus* as well as *Streptococcus* species belonging to the normal oropharyngeal microbiota. These bacterial species can simultaneously co-infect the lungs of cystic fibrosis (CF) patients (Sibley et al., 2008b, 2009, 2011) and dynamic interactions between these organisms have previously been demonstrated to alter *P. aeruginosa* virulence (Duan et al., 2003; Sibley et al., 2008a). In the scenario described below, we suggest that a single antibiotic therapy may affect the overall community structure and subsequently the antibiotic resistance of the community by initiating a cascade of interspecies interactions mediated by antibiotics and non-antibiotic metabolites (**Figure 5**).

Following an antibiotic treatment, many of the bacterial cells in the community will lyse releasing their intracellular content including eDNA and NO that could potentially induce antibiotic tolerance (Mulcahy et al., 2008; Gusarov et al., 2009). However, because of the population heterogeneity in terms of susceptibility and resistance/tolerance (persisters, stationary phase cells, aggregates, and biofilms) a large number of cells will die while some will survive. Over the course of the treatment, antibiotics will be present at different concentrations at different times and sites and therefore mediating different types of interactions with viable bacterial cells. At subinhibitory concentrations, antibiotics will induce different biological responses, for which antibiotic resistance/tolerance will subsequently be affected. In this particular polymicrobial community, β-lactams will induce the release of eDNA from *S. aureus* in an autolysin-dependent manner and triggering biofilm formation as a growth protective mechanism (Kaplan et al., 2012). At the same time, subinhibitory concentrations of other antibiotics will induce the production of pyocyanin in *P. aeruginosa* (Shen et al., 2008) resulting in the generation of H_2_O_2_, cell lysis, and subsequent release of more eDNA (Das and Manefield, 2012). Streptococci bacteria are known to generate H_2_O_2_ causing cell lysis and the release of eDNA (Shen et al., 2008). The accumulation of eDNA, through antibiotic and non-antibiotic interactions, will chelate cations and primarily the reduced cation concentrations that are directly sensed by the cells leading to antibiotic tolerance (Mulcahy et al., 2008). In addition, the community would favor biofilms and aggregates as modes of growth (Kaplan et al., 2012), which are generally more tolerant to antibiotics.

The concentration of antibiotics will gradually decrease over time allowing the remaining viable bacterial cells to grow back. The CF lung is rich in amino acids (Palmer et al., 2007) and actively growing bacteria will release ammonia as a by-product of amino acid catabolism. Ammonia sensing by bacteria within the community will induce the synthesis of polyamines leading to increased tolerance against ampicillin and tetracycline (Bernier et al., 2011) and oxidative stress (Bernier et al., 2011; Johnson et al., 2012). Further, the CF lung itself is rich in extracellular polyamines (Grasemann et al., 2012), which could directly affect the antibiotic tolerance profile of the community (El-Halfawy and Valvano, 2012) independently of ammonia sensing. Further, the consequences of these responses may impact syntrophic and other metabolite-mediated interactions that can further alter community composition.

For bacterial species that are naturally competent, the abundance of eDNA may also represent an excellent source of antibiotic resistance genes. Subinhibitory antibiotics induce the SOS response and competence systems (Charpentier et al., 2012) in Streptococci leading to horizontal gene transfer and the acquisition of resistance genes. Thereafter, the new-acquired resistance gene will be transferred vertically through bacterial division resulting in a new drug-resistant Streptococci strain. Further, mutation rates are also increased in all bacteria upon antibiotic exposure leading to the generation of potential new drug-resistant mutants. Resistance mechanisms that chemically inactivate antibiotics also have the potential to reduce the concentration to subinhibitory levels for susceptible cells in the community.

Through various inducible mechanisms mediated by antibiotics and non-antibiotic metabolites, the overall composition of the community may change over time. This complex network of interactions is not reflected in standard antibacterial susceptibility and these processes likely contribute to the frequent failure of antibiotics to reduce the population of susceptible organisms in patients. This will be more likely to occur in polymicrobial infections or when pathogens are part of a normal host community such as in upper respiratory or gastrointestinal infections.

## CONCLUSIONS AND PERSPECTIVES

The increase of antibiotic-resistant pathogens represents a very significant threat and challenge in the fight against infectious diseases. The complexity of the antibiotic resistome demonstrates that antibiotic resistance will always be a menace even for synthetic antibiotics. Fluoroquinolones represent a good example of synthetic drugs against which bacteria have quickly evolved resistance (Ruiz, 2003; D’Costa et al., 2006, 2007). More judicious use of antibiotics clinically and restricting non-medical applications may slow down the spread of drug-resistant bacteria and the emergence of new antibiotic-resistant pathogens, but the breadth of the antibiotic resistome and the capacity of microbes to rapidly evolve will make this an ongoing struggle. The chemical warfare that has been going on in microbial communities for hundreds of millions of years is something of a double-edged sword. Most antibiotics in use today have a microbial origin. Microbial secondary metabolites have also been a valuable source of drugs not restricted to just antibiotics. At the same time these communities have evolved complex resistance mechanisms that can rapidly spread from natural environments to the clinic.

Since most antibiotics are natural molecules involved in chemical interactions between bacteria in communities, it has become important to expand our understanding of the effects of these interactions on bacterial behaviors including antibiotic resistance. Because the presence of subinhibitory antibiotics can result in a phenotype, the responses are subject to evolution and natural selection, the same as toxic interactions. In this review, we have discussed and presented various scenarios on how antibiotics at subinhibitory concentrations have the potential to induce different biological responses leading to a general modification in the antibiotic resistance profiles of both environmental and host-associated bacteria. These non-lethal interactions can act as stress inducers or be sensed as cues by receiver bacteria. Activation of the SOS stress response by antibiotics appears to reduce the efficacy of antibiotic treatments and facilitate the evolution of resistance. Therefore, new therapeutic strategies targeting the SOS response may in return increase antibiotic efficacy. Blocking the LexA cleavage, therefore the SOS response, reduces the ability of *E. coli* to develop resistance toward ciprofloxacin and rifampicin both *in vivo* and *in vitro*, through mutations (Cirz et al., 2005). This suggests that suppressing the SOS response would inhibit mutation rate, which is an important downstream SOS-associated phenotype involved in bacterial evolution and antibiotic resistance.

Manipulating cellular physiology has the potential to enhance the efficacy of antibiotics even in resistant strains. A recent study explored this possibility and reported that an engineered bacteriophage targeting the SOS response network enhanced the killing efficacy of bactericidal antibiotics and survival in mice (Lu and Collins, 2009). The enhanced antibiotic killing by SOS-targeting phages was also effective against persister and biofilm bacteria (Lu and Collins, 2009).

The nature of the effect of volatile ammonia on antibiotic resistance is dependent on the class of antibiotic. While bacterial exposure to volatile ammonia induces tolerance against β-lactam and tetracycline antibiotics it increases the efficacy of aminoglycosides (Bernier et al., 2011). Interestingly, reactivation of the metabolism of *E. coli* persisters by the addition of various metabolites (glucose, mannitol, fructose, pyruvate) restored their sensitivity to aminoglycosides to a level comparable to non-persister cells (Allison et al., 2011). These two studies demonstrate the proof of concept that bacterial interactions occurring in natural communities via non-antibiotic molecules have the ability to increase antibiotic efficacy. Non-antibiotic metabolites like ammonia or glucose could be considered as antibiotic potentiators in synergistic drug combination therapies. The combinations of antibiotics and non-antibiotic drugs were showed to enhance antimicrobial efficacy against multidrug-resistant bacteria in both *in vivo* and *in vitro* suggesting that synergistic drug combinations have therapeutic potentials (Ejim et al., 2011).

Furthering our understanding of bacterial interactions mediated by antibiotics and non-antibiotic molecules will be valuable in the development of new strategies to combat antibiotic resistance. Although significant findings have been made in this emerging area of research, we need to further expand our views of these dynamic interactions in microbial communities. The conventional approach of determining *in vitro* susceptibilities to isolated organisms has many limitations clinically and in polymicrobial infections, many community interactions can reduce antibiotic efficacy in addition to traditional resistance mechanisms. Understanding chemical interactions within microbial communities should include the role of antibiotics in these communities and mechanisms of resistance. This will provide new opportunities and strategies to accelerate the discovery of bioactive molecules. Finally, we have to continue our efforts toward our global understanding of these non-classical views of antibiotic-mediated resistance mechanisms as complementary and potential strategies to new drug development programs in order to control and adequately manage the spread of antibiotic resistance in the future.

## Conflict of Interest Statement

The authors declare that the research was conducted in the absence of any commercial or financial relationships that could be construed as a potential conflict of interest.
